# Indocyanine green kinetics with near-infrared spectroscopy predicts cerebral hyperperfusion syndrome after carotid artery stenting

**DOI:** 10.1371/journal.pone.0180684

**Published:** 2017-07-12

**Authors:** Ichiro Nakagawa, Hun Soo Park, Shohei Yokoyama, Shuichi Yamada, Yasushi Motoyama, Young Su Park, Takeshi Wada, Kimihiko Kichikawa, Hiroyuki Nakase

**Affiliations:** 1 Departments of Neurosurgery Nara Medical University, Nara, Japan; 2 Departments of Radiology, Nara Medical University, Nara, Japan; Medical Photonics Research Center, Hamamatsu University School of Medicine, JAPAN

## Abstract

**Background:**

Cerebral hyperperfusion syndrome (HPS) is a potentially life-threatening complication following carotid artery stenting (CAS) and carotid endoarterectomy (CEA). Early prediction and treatment of patients at risk for HPS are required in patients undergoing CAS because HPS occurs significantly earlier after CAS than CEA. Near-infrared spectroscopy (NIRS) is often used for monitoring, and indocyanine green (ICG) kinetics by NIRS (ICG-NIRS) can detect reductions in cerebral perfusion in patients with acute stroke. However, whether ICG-NIRS can predict postoperative hyperperfusion phenomenon (HP) after carotid revascularization is unclear.

**Objective:**

Here, we evaluated whether the blood flow index (BFI) ratio calculated from a time-intensity curve from ICG-NIRS monitoring can predict HPS after CAS.

**Methods:**

The BFI ratio was prospectively monitored using ICG-NIRS in 135 patients undergoing CAS. Preoperative cerebrovascular reactivity (CVR) and the postoperative asymmetry index (AI) were also assessed with single-photon emission computed tomography before and after CAS, and the correlation was evaluated. In addition, patients were divided into two groups, a non-HP group (n = 113) and an HP group (n = 22), and we evaluated the correlation with hemodynamic impairment in the ipsilateral hemisphere and clinical results.

**Results:**

Twenty-two cases (16%) showed HP, and four (3%) showed HPS after CAS. The BFI ratio calculated from ICG-NIRS showed a significant linear correlation with preoperative CVR and postoperative AI (r = −0.568, 0.538, P < 0.001, <0.001, respectively). The degree of stenosis, the rate of no cross flow, preoperative CVR, and the incidence of HPS were significantly different between the groups.

**Conclusions:**

Measurement of ICG kinetics by NIRS is useful for detection of HPS in patients who underwent CAS.

## Introduction

Cerebral hyperperfusion syndrome (HPS) is defined as a major increase in ipsilateral cerebral blood flow (CBF) following revascularization for carotid artery stenosis with carotid artery stenting (CAS) and carotid endoarterectomy (CEA). HPS is a life-threatening complication that occurs in approximately 3–5% of patients after treatment.[1] When not recognized quickly and not treated adequately, HPS can lead to intracerebral hemorrhage, the most feared complication, which is associated with a mortality of 40%.[2] Furthermore, a recent study suggested that HPS occurs significantly earlier after CAS than after CEA.[3] Therefore, prediction and early treatment of patients at risk are essential in patients undergoing CAS. CAS is currently becoming widely performed as a less invasive alternative to CEA, and a recent randomized trial showed no differences in stroke, myocardial infarction, or death between CAS and CEA. However, CAS patients had a higher incidence of periprocedural stroke.[4]

Regional CBF (rCBF) can be measured with positron emission computed tomography (PET) and single-photon emission computed tomography (SPECT). CBF measurement is useful for predicting HPS following CAS and CEA[5]. However, PET and SPECT are expensive, complicated, and time-consuming procedures. Magnetic resonance (MR) perfusion and CT perfusion represent an alternative to non-invasive CBF measurement, however, these evaluations cannot be performed during CAS. [6, 7]

Near-infrared spectroscopy (NIRS) is currently often used for monitoring of frontal lobe regional cerebral O_2_ saturation following CAS and CEA, because this technique is a promising alternative for cerebral monitoring.[8–10] NIRS provides continuous measurement, including assessment of hemoglobin concentration changes and tissue oxygen index, and a high degree of sensitivity and specificity during carotid surgery.[11, 12] The amplitude of tissue oxygen index changes during CAS can predict postoperative HPS.[10]

Animal studies measuring CBF with an intravenous bolus of indocyanine green (ICG) and NIRS have shown a high correlation between absolute blood flow measurements obtained with the radioactive microsphere technique and the cerebral blood flow index (BFI) obtained noninvasively with ICG kinetics by NIRS (ICG-NIRS).[13] In patients, current studies have reported that ICG-NIRS detects perfusion reductions with acute middle cerebral artery (MCA) infarction.[14] However, whether ICG-NIRS can predict postoperative hyperperfusion phenomenon (HP) after carotid revascularization is unclear.

The aim of the present study was to evaluate whether the BFI ratio calculated from a time-intensity curve from ICG-NIRS monitoring predicts HP during CAS, and to investigate whether the BFI ratio is correlated with rCBF changes measured by SPECT before and after CAS.

## Patients and methods

### Inclusion criteria of patients

Between January 2012 and October 2016, 135 consecutive patients who underwent CAS for carotid artery stenosis at Nara Medical University were prospectively enrolled in the present study. The criteria used for CAS included stenosis >80% for asymptomatic lesions or stenosis >50% for symptomatic lesions, and patients with a high risk of CEA in accordance with the Stenting and Angioplasty with Protection in Patients at High Risk for Endarterectomy (SAPPHIRE) criteria.[15] All patients underwent preoperative angiography for evaluation of the degree of stenosis. No cross flow was diagnosed with preoperative angiography as the absence of visualization of an established anatomical segment of A1-4 for the anterior cerebral artery and M1-4 for the MCA by the Matas or Allcock maneuver. [15] MR imaging for plaque characterization was also performed on a 3-T system (Verio; Siemens). The signal intensities were measured with the region of interest drawn over the carotid plaque at the most severely stenotic level. The signal intensity ratio of the plaque relative to adjacent muscle was calculated, and unstable plaque was defined as >1.5, according to a previous report. [16] All procedures were performed under local anesthesia using a filter protection device. Dual-channel NIRS was used to measure cerebral oxygen saturation during and after the CAS procedure. The correlation between the BFI ratio and cerebrovascular reactivity (CVR) before CAS and asymmetry index (AI) after CAS was analyzed in all patients. These data were obtained from continuous NIRS monitoring and SPECT that was performed before and after the CAS procedure. All cases were classified into two groups, the non-hyperperfusion (non-HP) group and the hyperperfusion (HP) group according to previous studies.[17, 18] All patients signed a written authorization allowing access to their medical records for research purposes, and the research protocols were approved by the institutional review board of Nara Medical University (No. 459). All patients were provided with an informed consent document that explained all of the CAS procedures.

### NIRS

A transcranial cerebral oximeter (NIRO-200NX, Hamamatsu Photonics, Japan) was used to monitor the oxygen parameters. After thorough cleaning of the patient’s skin, the sensors were bilaterally and symmetrically placed over the forehead according to the manufacturer’s instructions. NIRS monitoring was performed from puncture to 24 hours after CAS. It was continued until 7 days after CAS if HPS occurred. The oximeter uses a method that combines the multi-distance measurements of optical attenuation. The underlying mathematical model is based on spatially resolved spectroscopy and the modified Beer-Lambert law. The NIRO-200NX generates three wavelengths (735, 810, 850 nm) of infrared light that are produced by a light-emitting diode. Using one emitting laser diode and two detecting photodiodes, we were able to measure the ratio of oxygenated hemoglobin to total hemoglobin.

### ICG-NIRS

**Fig 1:**
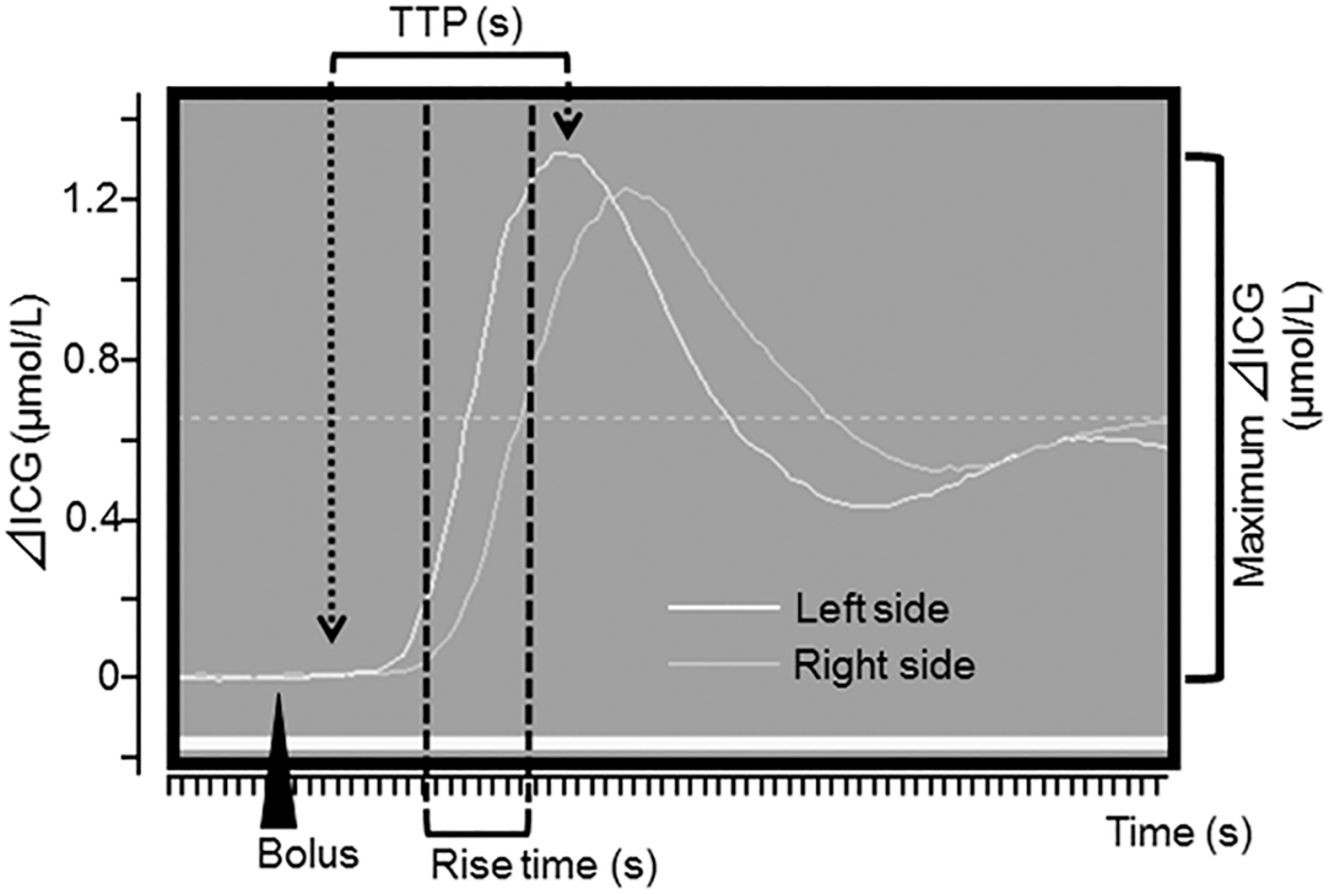
Typical time-intensity curve from indocyanine green (ICG) measurement in patient with carotid artery stenosis. TPP; time to peak.

ICG was injected rapidly into the central venous line with a mean dosage of 0.2 mg/kg body weight. Absolute concentration changes of ICG were calculated from light attenuation according to the modified Beer-Lambert law using specific software (Hamamatsu Photonics, Hamamatsu, Japan). We assessed the time to peak (TPP; the time interval between 0% and 100% of the maximum signal), the maximum ICG concentration (μmol/l), the rise time (defined as the time between 10% and 90% of the ICG maximum), and BFI (maximum ΔICG/rise time). The interindividual difference in ICG kinetics between the two hemispheres was also calculated. These parameters were semi-automatically calculated from the ICG time-intensity curve by N200NX ICG Analyze software (Hamamatsu Photonics). A typical measurement is shown in Fig 1. ICG kinetics was measured before puncture, just after CAS, and 6 and 24 hours after CAS. As BFI is semiquantitative measure of CBF, BFI ratio was evaluated for intraindividual differences. [14, 19] The BFI ratio was defined as BFI just after CAS/BFI before puncture.

### SPECT

All patients underwent SPECT using a rotating three-head gamma camera (GCA-9300A; Toshiba Medical Systems, Tochigi, Japan) before and after the procedure as perioperative management for HPS. CBF in the cerebral hemisphere was quantified using ^123^I-SPECT within 2 weeks before and the day after the procedure. The details of the IMP SPECT study with acetazolamide (ACZ) challenge have been reported.[20, 21] The absolute relative CBF values were quantified with the microsphere method. An irregular, mirror-shaped region of interest was placed bilaterally in the entire cerebral cortex at the level of the parietal lobe, excluding any infarcts, and in the corresponding contralateral region. Resting rCBF, CVR to ACZ challenge, and AI were quantitative measured for the hemodynamic reserve. The resting rCBF control value obtained from 20 heathy volunteers (18 men and 2 women; aged 65 to 85 years old; mean age, 76.3 years old) was 30.6 ± 2.9 ml/100 g/min. A resting rCBF value lower than the mean + 2 SD (i.e., 24.8 ml/100 g/min) was defined as a decrease in resting rCBF. The CVR (%) was calculated as follows: (ACZ challenged rCBF in the affected cerebral hemisphere–resting rCBF in the affected territory)/resting rCBF in the affected territory ×100%. The control CVR value obtained from 20 heathy volunteers (18 men and 2 women; aged 68 to 85 years old; mean age, 76.3 years old) was 50.4 ± 13.3%. A CVR value lower than the mean + 2 SD (i.e., 23.8%) was defined as reduced CVR. The AI (%) was calculated as the measure of blood flow between the two cortical hemispheres by taking the ratio of rCBF of the affected to the unaffected hemisphere, excluding any ischemic/infarcted areas (rCBF in the affected cerebral hemisphere/rCBF in the mirror territory) ×100. The control AI value obtained from 12 heathy volunteers (11 men and 1 woman; aged 65 to 85 years old; mean age, 76.7 years old) was 102.1 ± 0.85%. An AI value higher than the mean + 3 SD (i.e., 104.6%) was defined as an increase in AI. In the present study, hyperperfusion after CAS was defined as a rCBF increase of >4.6% compared with that on the normal side.

### Interventional procedure

During the CAS procedure, which was performed under local anesthesia, intravenous heparin was used to maintain an activated clotting time of >275 s. An 8-Fr guiding catheter was first placed in the common carotid artery. After crossing the stenotic lesion with a guidewire, a filter-type embolic protection device (FilterWire EZ; Stryker, Kalamazoo, MI) was inserted distal to the stenotic lesion. Following pre-dilation of the lesion with a 3- or 4-mm diameter balloon, the stent (Carotid Wallstent; Stryker, Precise; Johnson & Johnson, New Brunswick, NJ, or PROTÉGÉ; Covidien, Dublin, Ireland) was deployed. Using angioplasty balloons with diameters no greater than approximately 80% of the normal luminal diameter distal to the stenosis, conservative post-dilation was then performed. The procedure was monitored with intravascular sonography (Eagle Eye Gold: Volcano Corporation, San Diego, CA). At the end of the procedure, the embolic protection device was removed. During patient follow-up, all symptomatic ischemic events, hemorrhagic events within 30 days, and any abnormalities on magnetic resonance imaging (MRI) diffusion-weighted images (DWI) that occurred were recorded.

### Clinical diagnosis of cerebral HPS

A diagnosis of HPS required the following: 1) Occurrence of headache, seizure, confusion, deterioration of consciousness level, and/or development of focal neurological signs such as motor weakness, 2) absence of any additional ischemic lesion on MRI performed the first postoperative day, and 3) postoperative increase in rCBF in the ipsilateral hemisphere exceeding the flow in the contralateral hemisphere as measured using SPECT.

### Data analysis and statistics

Measurements for each group are expressed as means ± standard deviation. Comparisons between two groups were assessed using the Mann-Whitney U test, Fisher’s exact test, ANOVA test. Pearson correlation test were used to examine the relationship between preoperative CVR, postoperative AI and BFI ratio. Differences were deemed statistically significance if P < 0.05.

## Results

### Study population

**Fig 2:**
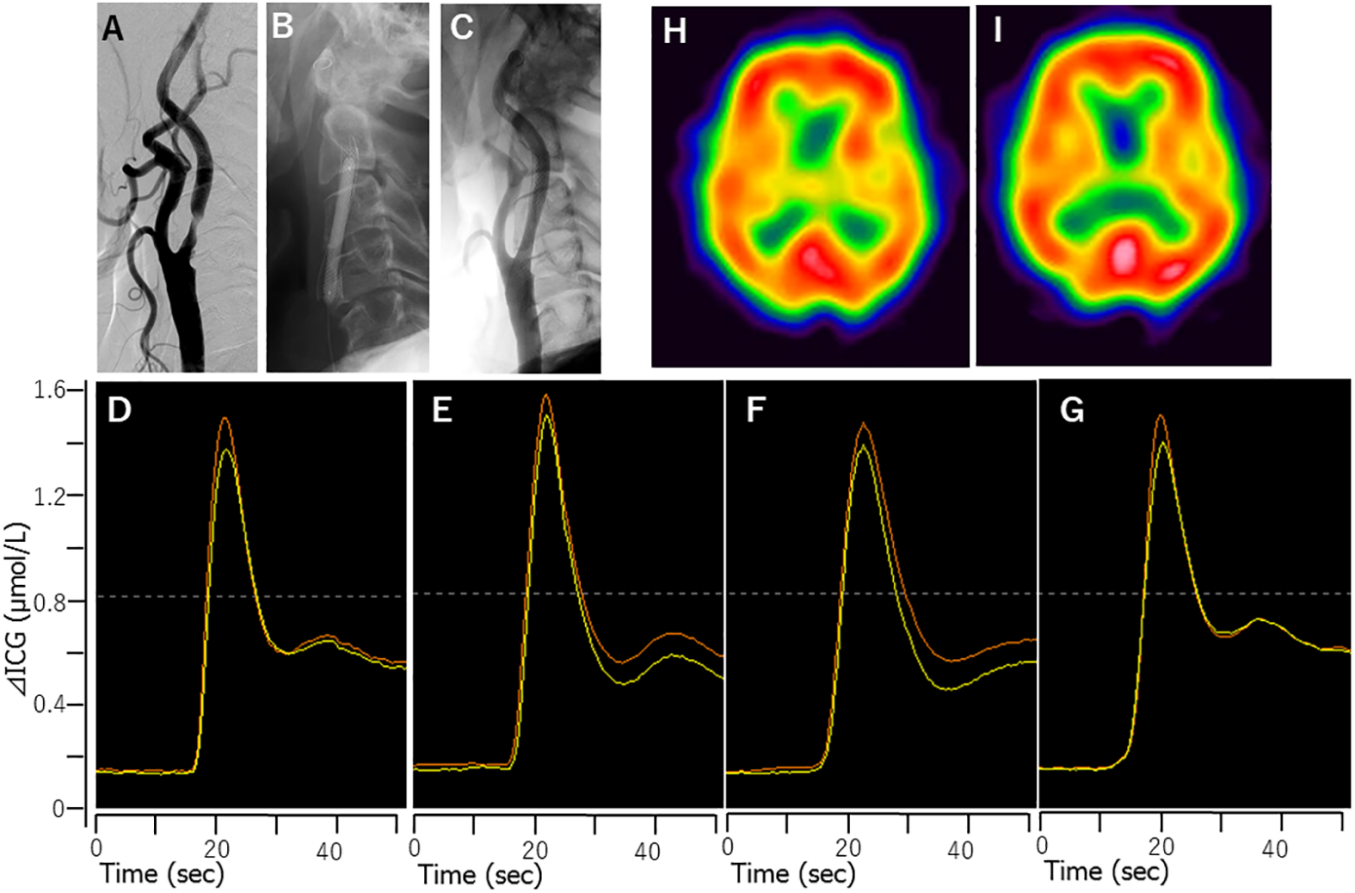
Case 1 (non-HP): A 64-year-old male presented with severe symptomatic right internal carotid artery stenosis. Right common carotid angiography showed severe right internal carotid artery stenosis (A). Balloon angioplasty for post-dilatation was performed after stent placement with filter protection (B). Right common carotid angiography after CAS confirmed stent patency (C). Time-intensity curve from ICG-NIRS monitoring before (D), just after (E), 6 hours after (F), and 1 day after CAS (G). No remarkable change was seen in the time-intensity curve time course throughout the measurement. Yellow curve; affected side, Orange curve; unaffected side. We found no remarkable differences between preoperative rCBF in the right hemisphere with ^123^I-IMP SPECT in the resting state between before (H) and 1 day after CAS (I). MRI showed no additional ischemic lesions. He was discharged without any neurological deficits.

**Fig 3:**
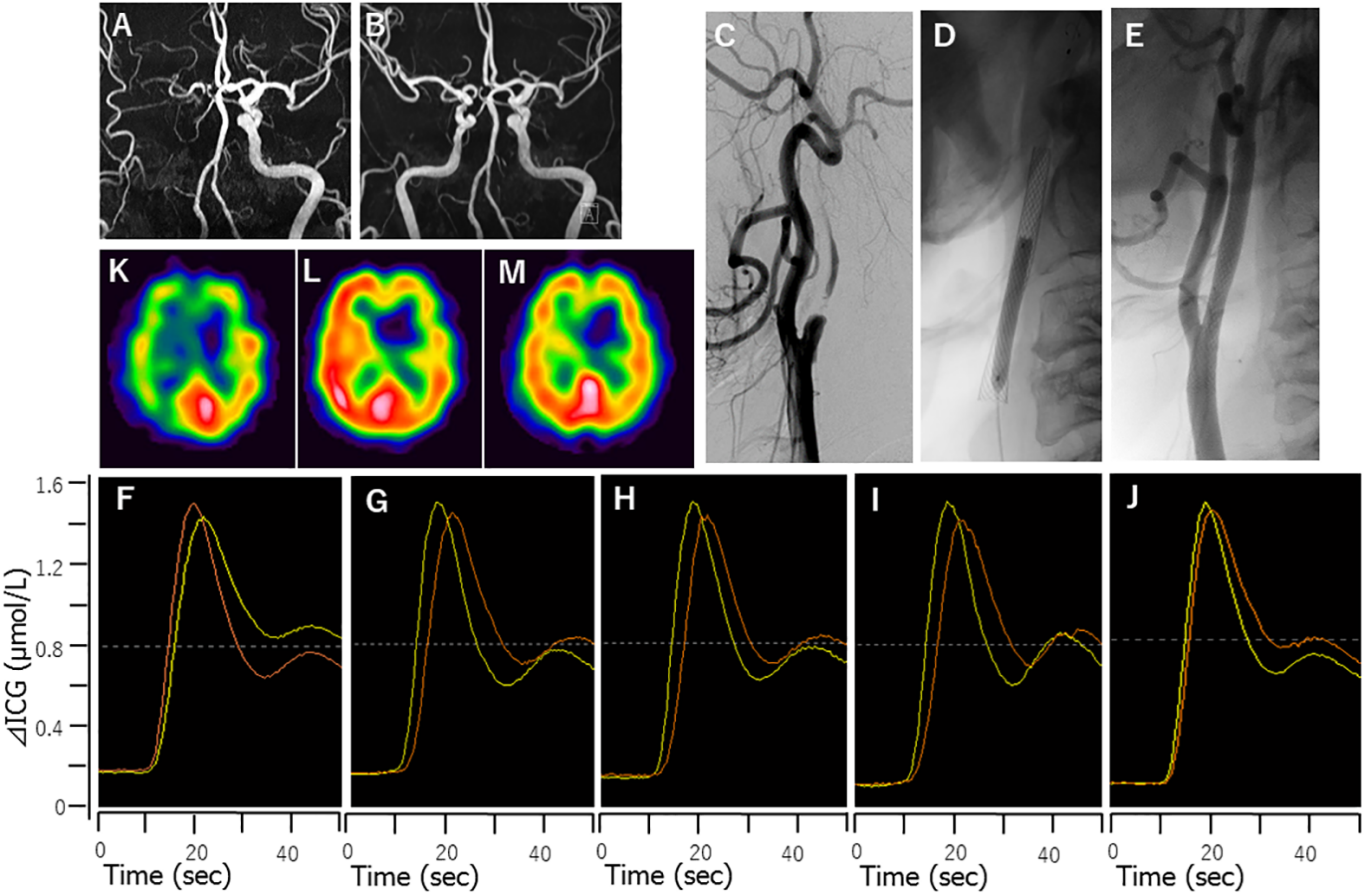
Case 2 (HPS case): A 70-year-old male presented with symptomatic right internal carotid artery stenosis. Magnetic resonance angiography showed reduction in the right internal carotid artery signal before (A) and after CAS (B). He underwent right-side CAS with NIRS monitoring. Right common carotid angiography showed right internal carotid artery nearly occlusion (C). Balloon angioplasty for post-dilatation was performed after stent placement with filter protection (D). Right common carotid angiography after CAS confirmed stent patency (E). MRI showed no additional ischemic lesions, and hence, he was diagnosed with postoperative HPS. The patient’s systolic blood pressure was strictly controlled between 100–140 mmHg, and sedation was induced with dexmedetomidine. Seven days after CAS, he recovered from the symptoms and he was discharged without any neurological deficits. Time-intensity curve from ICG-NIRS monitoring before (F), just after (G), 6 hours after (H), 1 day after (I), and 7 days after CAS (J). A remarkable change in the time-intensity curve time just after CAS was observed; the difference disappeared 7 days after CAS. Yellow curve; affected side, Orange curve; unaffected side. Preoperative ^123^I-IMP SPECT in the resting state showed a decrease in rCBF in the right hemisphere (K). One day after CAS, hyperperfusion was seen in the ipsilateral hemisphere (L). One week after CAS, hyperperfusion in the ipsilateral hemisphere had normalized (M).

This study enrolled and evaluated a total of 135 patients (118 males and 17 females; mean age, 74.5 years old, age range; 41–91 years old). The lesion characteristics including the average degree of stenosis by NASCET, the rate of symptomatic lesions, and the rate of vulnerable plaques were 81.4 ± 11.0%, 51%, and 56%, respectively. In CAS treatment, 77% of cases were treated with a closed-cell type stent, and 13% of all patients demonstrated a bright DWI lesion a day after CAS. Patient’s systolic blood pressure was strictly controlled between 100–140 mmHg before, during and after CAS. No patients developed ischemic or hemorrhagic complications after CAS. The mean preoperative CVR and postoperative AI measured by ^123^I-IMP SPECT were 41.9 ± 23.1% and 100.2 ± 6.6%, respectively. The relationship between preoperative CVR and postoperative AI is shown in Fig 1. Twenty-two cases (16%) showed HP (postoperative AI >104.6%) after CAS. Four cases (3%) showed HPS after CAS. Typical BFI course, SPECT, and angiographic findings for patients without and with HPS are shown in Figs 2 and 3, respectively.

### Relationship among the BFI ratio, CVR, and AI

**Fig 4:**
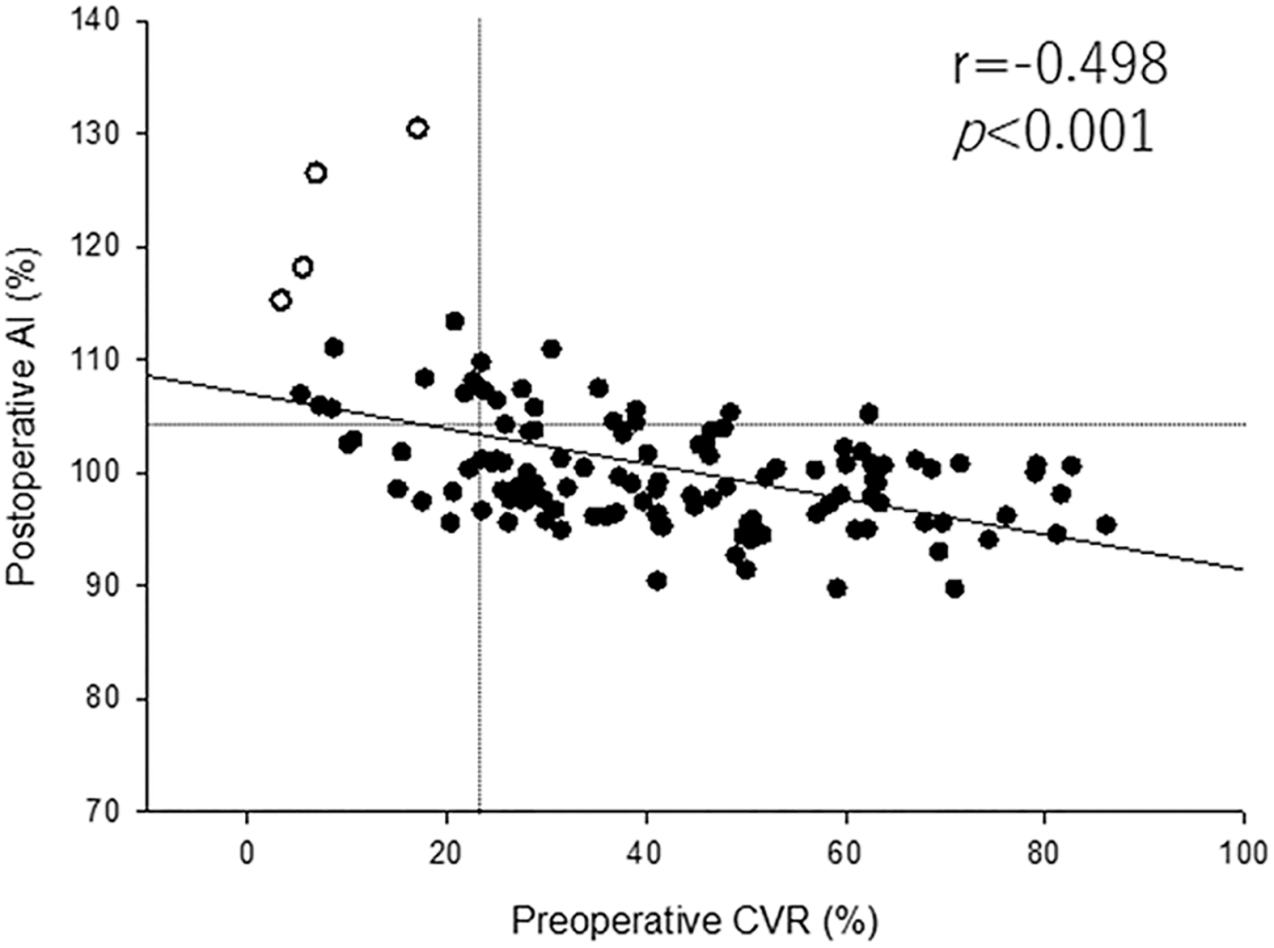
Preoperative cerebrovascular reactivity (CVR) and postoperative asymmetry index (AI) the day after stent placement. A CVR value lower than the mean + 2 SD (i.e., 23.8%) was defined as reduced CVR (vertical line), and an AI value higher than the mean + 3 SD (i.e., 104.6%) was defined as increase in AI (horizontal line). Open circles indicate patients with HPS after CAS.

**Fig 5:**
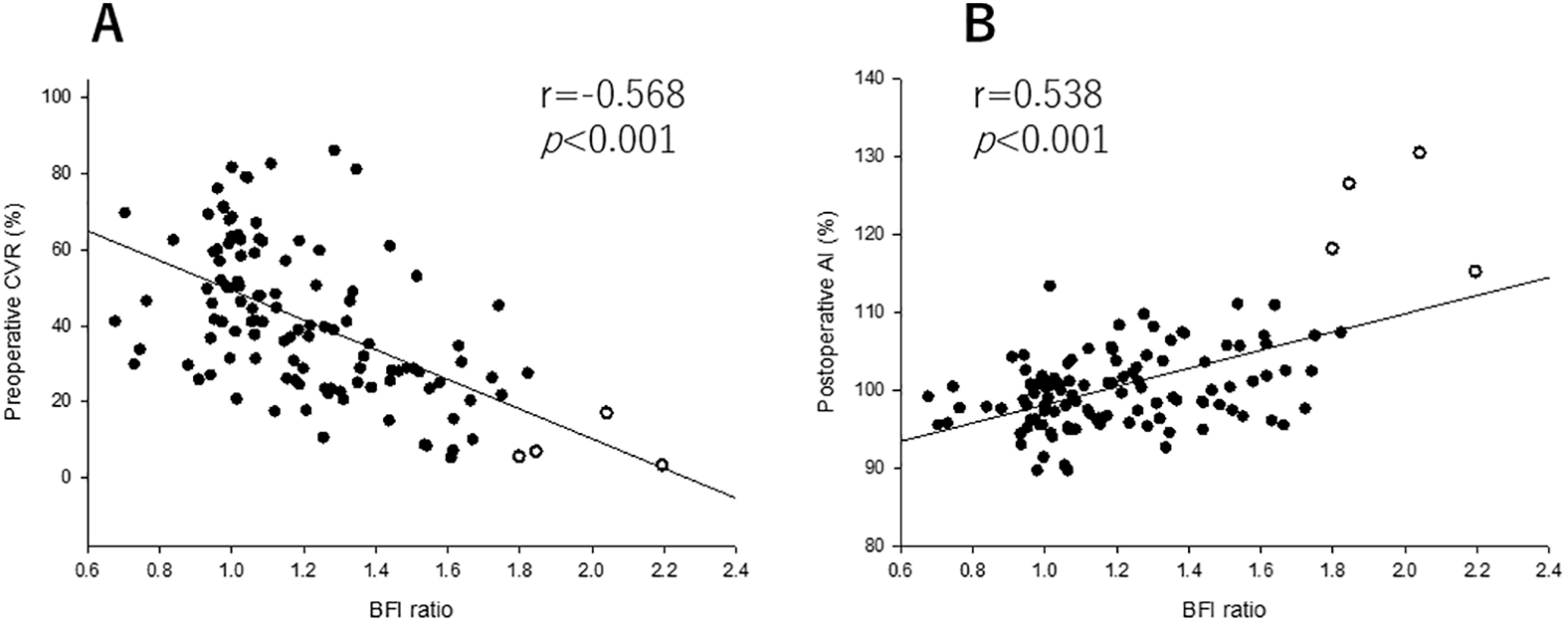
The BFI ratio was correlated with preoperative CVR (A). The BFI ratio was also correlated with the postoperative AI (B). Open circles indicate patients with HPS after CAS.

The relationship between preoperative CVR and postoperative AI is shown in Fig 4. A significant linear correlation was observed between these parameters (r = −0.498, P < 0.001). Twenty of 135 patients showed reduced CVR (CVR <23.8%) in the ipsilateral hemisphere before CAS, and 22 of 135 patients showed HP (AI >104.6%) after CAS. Four patients demonstrated HPS after CAS (white circles in Fig 4A). The BFI ratio calculated from ICG-NIRS showed a significant linear correlation with preoperative CVR and postoperative AI (r = −0.568, 0.538, P < 0.001, <0.001, respectively) (Fig 5A and 5B).

### Comparison between patients with and without HP after CAS

Patients were divided into two groups according to postoperative AI after CAS as described above. Table 1 presents the clinical characteristics of the patients in the two groups.

**Table 1:** Patient and lesion characteristics.

	No. of Patients (%)		
Variable	Non-Hyperperfusion group	Hyperperfusion group	P value
	(n = 113)	(n = 22)	
General characteristics			
Mean age, years (range)	75 (41–91)	74 (58–86)	0.816
Females	14 (12%)	3 (14%)	0.134
Risk factor			
Hypertension	86 (76%)	20 (91%)	0.207
Diabates	33 (29%)	9 (41%)	0.212
Current smoker	29 (26%)	2 (9%)	0.157
Chronic kidney disease	46 (41%)	13 (59%)	0.175
Medications			
Statins	113 (100%)	22 (100%)	1.000
Angiotensin receptor blockers	69 (61%)	12 (55%)	0.739
Proton pump inhibitor	15 (13%)	9 (23%)	0.416
Lesion characteristics			
Symptomatic	60 (53%)	9 (41%)	0.416
Vulnerable plaque	64 (57%)	11 (50%)	0.735
Degree of stenosis (%)	80.4 ± 11.3	86.5 ± 7.8	0.029*
No cross flow	9 (8%)	5 (21%)	<0.001*
Preoperative CVR	46.1 ± 22.3	23.6 ± 17.0	<0.001*
Postoperative AI	98.0 ± 3.9	110.4 ± 6.8	<0.001*

CVR; cerebrovascular reactivity, AI; asymmetry index

*: *p*<0.05

For the non-HP group (113 patients), the mean age was 75 years old (range, 41–91 years old), and for the HP group (22 patients), the mean age was 74 years old (range, 58–86 years old). All of the baseline characteristics were similar between the groups. There were no significant differences between the two periods in the risk factors (hypertension, diabetes, current smoker, and chronic kidney disease) and medications (statins, angiotensin receptor blockers, and proton pump inhibitors). The lesion characteristics including the rate of symptomatic lesions and the rate of vulnerable plaques were similar between the two groups. However, the average degree of stenosis by NASCET, the rate of no cross flow, preoperative CVR, and postoperative AI were significantly different between the groups.

All 135 patients underwent CAS successfully. We observed no significant differences between the two groups for the embolic protection method, the stent design, bright DWI lesions 1 day after CAS, or ischemic/hemorrhagic events 30 days after CAS (Table 2). However, the incidence of HPS was significantly higher in the HP group than the non-HP group (P < 0.001) (Table 2).

**Table 2:** Treatment and clinical results.

	No. of Patients (%)		
Variable	Non-Hyperperfusion group	Hyperperfusion group	P value
	(n = 113)	(n = 22)	
Treatment			
Filter protection	113 (100%)	22 (100%)	1.000
Open cell stent	27 (23.9%)	4 (18.2%)	0.760
Clinical results			
DWI positive	14 (24%)	4 (18%)	0.849
Ischemic events	0 (0%)	0 (0%)	1.000
Hemorrhagic events	0 (0%)	0 (0%)	1.000
Hyperperfusion syndrome	0 (0%)	4 (18%)	<0.001*

DWI; Diffusion Weighted image.

*: *p*<0.05

HPS was observed in four patients presenting with headache and confusion after CAS. However, intracranial hemorrhage did not occur because of postoperative sedation and strict blood pressure control immediately after suspicion of HPS. These four patients were discharged from our hospital without any neurological deficits.

Table 3 shows the comparison of ICG kinetics between the groups. BFI just after CAS and the BFI ratio in the HP group were significantly higher than those in the non-HP group (P = 0.002, <0.001, respectively).

**Table 3:** Comparison of ICG kinetics between the groups.

	No. of Patients (%)		
Variable	Non-Hyperperfusion group	Hyperperfusion group	P value
	(n = 113)	(n = 22)	
Before CAS			
Rise time (sec)	3.71±1.67	3.25±1.41	0.542
Maximum ΔICG (µmol/L)	0.98±0.28	0.82±0.32	0.097
BFI (μmol/L/sec)	0.31±0.11	0.28±0.07	0.356
Time to peak (sec)	11.61±1.98	12.39±1.96	0.100
Just after CAS			
Rise time (sec)	3.16±0.56	2.55±0.98	0.148
Maximum ΔICG (μmol/L)	0.94±0.29	1.04±0.34	0.130
BFI (μmol/L/sec)	0.34±0.10	0.42±0.10	0.002*
Time to peak (sec)	11.14±1.80	10.38±1.65	0.074
BFI ratio	1.15±0.23	1.51±0.31	<0.001*

ICG; Indocyanine green. BFI; Blood flow index.

*: *p*<0.05

**Fig 6:**
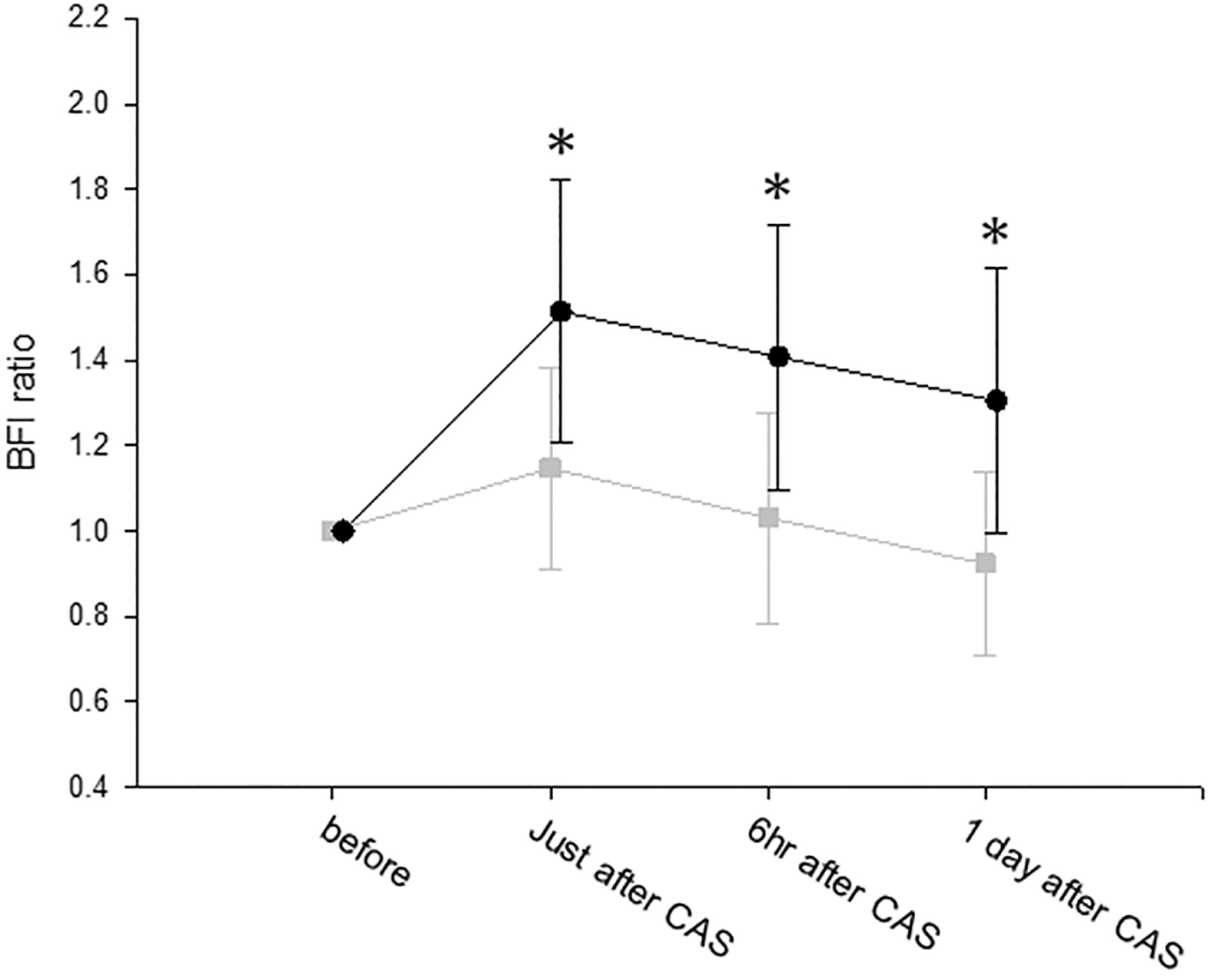
The time course of BFI between the hyperperfusion (HP) group and the non-hyperperfusion (non-HP) group. Black circles indicate the HP group, and gray squares indicate the non-HP group. *P < 0.05.

Fig 6 shows the time course of the change in the BFI ratio after CAS between the groups. We observed a significantly higher BFI ratio in the HP group at 6 hours and 1 day after CAS (P<0.05).

## Discussion

In the present study, we demonstrated that the BFI ratio, which were calculated from ICG-NIRS, showed significant linear relationships between not only preoperative CVR but also postoperative AI. Furthermore, in four patients with HPS after CAS, the BFI ratio was higher than those in non-HP patients. These results suggested that the measurement of ICG kinetics with NIRS provides a useful tool for detection of HPS in patients who underwent CAS.

Cerebral HPS after CEA or CAS is rare. However, severe complications such as intracranial hemorrhage and HPS are reported to be .2% to 18.9% in the literature.[1, 9] Recent reports have demonstrated that HPS occurs earlier after CAS (within 12 hours) than CEA.[3] Therefore, prediction and early treatment of patients at risk for HPS are essential, and assessment of the real-time situation with perioperative CAS is necessary. The efficacy of SPECT and PET for diagnosing postoperative HPS after CEA has been established.[22, 23] Ogasawara et al. demonstrated that preoperative measurement of ACZ-induced changes in rCBF, which is performed using SPECT scanning, can be used to identify patients at risk for hyperperfusion after CEA. In addition, post-CEA monitoring of rCBF performed using SPECT scanning results in timely and reliable identification of patients at risk for HPS.[23] Although SPECT and PET studies can accurately measure rCBF, they are expensive, complicated and time-consuming. MR perfusion and CT perfusion represent an alternative to non-invasive CBF measurement, however, these evaluations cannot be performed during CAS. [6, 7] Various alternative bedside monitoring methods have been described previously. NIRS is noninvasive, safe, and simple for measuring tissue oxygenation and NIRS is advantageous because it can be used to evaluate continuous, real-time changes in regional cerebral tissue oxygenation values.[11, 24]^,^ [10] However, the main disadvantage of NIRS is that it only allows monitoring of the relative concentration of oxyhemoglobin and cannot measure rCBF.

In contrast, ICG is a strong near-infrared absorber that has been used with NIRS to measure rCBF. [13, 25] In piglets, Kuebler et al. have shown that CBF can be estimated from the initial rise of the increasing dye concentration following application of a bolus of ICG.[13] Several attempts of bedside monitoring to access CBF by combining ICG and NIRS have been undertaken in piglets,[26] naonates,[27] and adults.[14, 28] Keller et al. proposed new algorithms to determine CBF as absolute numbers in ml/100g/min have been developed, which might allow to identify critical thresholds for cerebral ischemia detection.[25] Furthermore, Hopton et al. proposed a method for quantitative monitoring of cerebral blood volume by NIRS with ICG, simultaneously measuring the concentration of ICG in peripheral blood.[29] However, the absolute quantification of CBF by NIRS with ICG has proved difficult because of the unknown optical path length of photons through biological tissue.[25, 29] Recently, quantitative time-resolved NIRS has developed in clinical use.[30, 31] It will be possible to measure absolute quantification of CBF by time-resolved NIRS with ICG.

### The BFI and postoperative hyperperfusion

Reduced preoperative CVR to ACZ is a significant predictor of hyperperfusion after CEA and CAS.[5, 23, 32] Suga et al. observed post-CEA hyperperfusion in 12 (52%) of 23 patients with reduced preoperative CVR to ACZ, but in none of the patients with normal preoperative CVR (n = 67).[33] Buczek et al. reported that more patients with postoperative HPS had impaired CVR before the procedure compared with patients without postoperative HPS (63.6 vs. 26.5%); the sensitivity and specificity of CVR for HPS were 63.6 and 73.5%, respectively.[32] However, patients with reduced preoperative CVR do not always develop hyperperfusion after CEA or CAS. The incidence of HPS after CAS in patients with reduced preoperative CVR was 20% in the present study (Fig 4A), and these results were consistent with previous reports.[8]^,^ [17] In addition, patients with elevated postoperative AI did not always show reduced CVR preoperatively in the present study (Fig 4A). This could explain why rCBF on the affected side may be overestimated compared with rCBF on the contralateral side because of existing bilateral carotid steno-occlusive disease.

In contrast, an elevated BFI ratio calculated from ICG-NIRS were significant predictors of HPS in the present study (Fig 4B). Terborg et al. investigated cerebral perfusion values including BFI calculated from ICG-NIRS in patients with acute infarction in the territory of the MCA.[14] Patients with MCA territory infarction showed decreased BFI at the site of the MCA territory infarction compared with the unaffected hemisphere. The authors concluded that ICG-NIRS can detect perfusion reduction in patients with acute ischemic stroke. In addition, a recent report suggested that cerebral perfusion assessed with ICG-NIRS in patients with MCA territory infarction correlated well with values obtained with perfusion MRI.[19] These results were consistent with our results. Furthermore, the BFI ratio calculated from ICG-NIRS indicated a significant linear relationship between not only preoperative CVR but also postoperative AI (Fig 4C and 4D). These results suggested that cerebral perfusion assessed with ICG-NIRS can detect hyperperfusion after CAS as an alternative to PET and SPECT.

### Comparison of ICG kinetics between the non-HP and HP groups

Twenty-two cases (16%) showed HP (postoperative AI >104.6%) after CAS in the present study, and we observed significant differences in the degree of stenosis, no cross flow, preoperative CVR, and incidence of HPS between the groups (Table 1). Furthermore, the time course of the BFI ratio was significantly different between the groups after CAS (Fig 6). The presence of severe hemodynamic impairment due to unilateral steno-occlusive disease is associated with reduced CVR, not only in the hemisphere ipsilateral to the steno-occlusion but also because of collateral flow from the contralateral hemisphere.[34] A recent study using computational modeling of the cerebral circulation suggested that the diameters of the cerebral communicating arteries and the severity of carotid artery stenosis both have a significant influence on post-CAS cerebral hyperperfusion rates.[35] The present results are consistent with previous reports.

On the other hand, we found no significant differences between the HP and non-HP groups in absolute parameters including rise time, maximum ΔICG and TTP calculated from ICG kinetics before and after CAS, although we did find significant differences in the BFI ratio between the groups. Assessment of cerebral hemodynamics with ICG-NIRS yields time-intensity curves of cerebral perfusion that are independent of oxyhemoglobin. However, the absolute quantification of rCBF with ICG-NIRS has proven difficult due to the unknown optical path length of photons through biological tissue, scattering losses, and the influence of extracerebral tissue. Skin blood flow contaminates cerebral NIRS measurement, especially if the sensors are placed in the temporal region. This may explain the lack of correlation between ICG kinetics and rCBF measurement in a previous report.[36] In contrast, another report showed that the intravenous ICG bolus arrives early in cerebral tissue and is delayed in superficial layers.[37] The first part of the curve used for determining ICG kinetics mainly represents rCBF and is less contaminated by extracerebral tissue, especially forehead skin blood flow as in our setting. Therefore, the BFI ratio calculated from ICG-NIRS were correlated with CVR and AI from SPECT in the present study.

### Limitations

Our study has several limitations. The coverage area of NIRO-200NX was limited to the cortical region of the frontal lobe, because of the placement of oximeter sensors over the forehead. Furthermore, extracranial contamination such as extracranial blood flow, cerebral metabolism, arterial saturation, and hematocrit influence cerebral oxygen measured with NIRS. Skin blood flow usually contributes around 5% to NIRS measurements even increasing optode spacing (5cm) reduced surfice contribution.[38] In addition, because each wavelength of infrared light cannot be measured, we need to perform comparisons with the mean light length. However, this equipment may reflect relative hemodynamic changes. Preoperative cerebral microbleeds on T2* weighted MR image did not examine in the present study. No hemorrhagic complication was shown in the study, however, preoperative cerebral microbleeds may influence the incidence of hemorrhagic complication after CAS. Recent study suggested staged angioplasty can prevent HP after CAS. [39] Although additional intervention is needed, staged angioplasty may be considered a relatively simple and effective method to avoid HP in patients at high risk of HP after CAS. The study design was a nonrandomized study, and the small sample size may have introduced biases regarding patients and data collection. In addition, only four patients presented with HPS. Additional well-designed randomized controlled trials involving a large number of patients are required to confirm these results.

## Conclusions

The BFI ratio calculated from ICG-NIRS indicated a significant linear relationship between not only preoperative CVR but also postoperative AI. Furthermore, in four patients suffering from HPS after CAS, the BFI ratio was higher than those of non-HP patients. These results suggested that measurement of ICG kinetics with NIRS provides a useful tool for detection of HPS in patients who underwent CAS.

## Supporting information

S1 File ICG NIRS dataset.xlsx. (XLSX)
